# Disruption of the S1/S2 multibasic cleavage site attenuates infectious bronchitis virus, while S2′ partially restores viral virulence and expands tissue tropism

**DOI:** 10.1128/jvi.00614-26

**Published:** 2026-07-06

**Authors:** Ye Zhao, Jun Zhao, Yingfei Li, Linqing Duan, Rong Liang, Min Huang, Jing Zhao, Guozhong Zhang

**Affiliations:** 1 State Key Laboratory of Veterinary Public Health and Safety, College of Veterinary Medicine, China Agricultural University 34752, Beijing, China; 2 Key Laboratory of Animal Epidemiology of the Ministry of Agriculture, College of Veterinary Medicine, China Agricultural University 34752, Beijing, China; The University of Texas Southwestern Medical Center, Dallas, Texas, USA

**Keywords:** IBV, multibasic cleavage site, S1/S2, S2′, pathogenicity, tissue tropism

## Abstract

Multibasic cleavage motifs play critical roles in envelope glycoprotein-mediated viral entry by serving as substrates for proprotein convertases like furin. The gamma-coronavirus infectious bronchitis virus (IBV) inherently possesses a conserved S1/S2 multibasic cleavage site (MBCS) within its S gene. Notably, the IBV Beaudette strain uniquely harbors an additional S2′ MBCS, which extends its cellular tropism. Our previous work demonstrated that introducing the S2′ MBCS into the YN strain contributes to central nervous system damage. However, the functional interplay between the S1/S2 and S2′ MBCS has not yet been elucidated. Herein, we rescued several recombinant IBV mutants based on two different genotypes, engineered with distinct S protein cleavage patterns. Our results revealed that deletion of the S1/S2 MBCS results in a drastic reduction of viral replication efficiency and pathogenicity. This negative effect can be partially compensated by introducing the S2′ MBCS. The S2′ MBCS also rendered increased mortality and elevated viral loads in neurological organs—effects that were diminished with less efficient S1/S2 cleavage. Inconsistencies exist regarding the impact of MBCS mutations in different IBV strain backgrounds, which are mainly due to their differential dependence on host proteases, correlating with their distinct tissue tropism. Our study demonstrated a proteolytic coordination model for IBV S protein activation: The S1/S2 MBCS primarily confers efficient replication competence; the S2′ site acts as an amplifier of organ-specific pathogenicity, particularly enhancing neurotropism. This synergistic relationship is conserved across different IBV strains.

**IMPORTANCE:**

Multibasic cleavage sites (MBCSs) are critical determinants of coronavirus pathogenicity and tissue tropism. While infectious bronchitis virus (IBV) inherently possesses a conserved S1/S2 cleavage site, the functional significance of this site and its potential interplay with the unique S2′ MBCS found in the Beaudette strain remain poorly understood. Our study definitively establishes the critical role of the S1/S2 cleavage site for efficient IBV replication *in vitro* and full virulence *in vivo*, while the S2′ site can only partially restore viral virulence. The pathogenic enhancement conferred by the S2′ site is due to the heightened viral loads and enhanced organ-specific neurological damage. These findings provide essential insights into IBV pathogenesis governed by spike protein cleavage and establish a vital theoretical foundation for designing rationally attenuated vaccines and effective control strategies against avian coronaviruses.

## INTRODUCTION

Coronaviruses are taxonomically divided into four genera: *Alphacoronavirus*, *Betacoronavirus*, *Gammacoronavirus*, and *Deltacoronavirus*. Avian infectious bronchitis virus (IBV) belongs to the genus *Gammacoronavirus*, under the order Nidovirales, family Coronaviridae, and subfamily Orthocoronavirinae (1–3). All coronaviruses share a similar genome structure of a single-stranded, positive-sense RNA, approximately 26–32 kilobases in length. This RNA genome encodes several structural and non-structural proteins that are crucial for viral replication and pathogenesis. Among these proteins, the spike (S) protein plays a pivotal role in viral entry into host cells by binding to specific cellular receptors. The genetic diversity and mutation rate of coronaviruses contribute to their ability to adapt and cause emerging and re-emerging infections in humans and animals.

Multibasic cleavage sites (MBCSs) are critical elements in the biology of coronaviruses, particularly in viral infectivity and pathogenicity (4). The presence of the MBCS, which contains multiple arginine residues, allows for efficient cleavage by ubiquitously expressed furin and related proprotein convertases. The spike protein mediates the entry of the coronavirus into host cells, and the cleavage at the S1/S2 junction by host proteases like furin is a key step in this process. This cleavage is essential for the spike protein to undergo the necessary conformational changes that allow the virus to fuse with the host cell membrane and initiate infection. The acquisition of an MBCS in the spike protein of SARS-CoV-2 is considered a pivotal evolutionary step that enhanced the virus’s infectivity and transmission among humans (5, 6). In addition to SARS-CoV-2, other coronaviruses and even some influenza viruses have been shown to possess similar MBCS, which contribute to their pathogenicity. For instance, the H7N9 avian influenza virus evolved from a low pathogenic to a highly pathogenic form through the acquisition of an MBCS in its hemagglutinin protein, highlighting the broader significance of these motifs in viral evolution and disease emergence (7).

IBV is responsible for causing infectious bronchitis (IB) in chickens, an acute and highly contagious respiratory disease (8). Chickens are the primary natural hosts for IBV, and strains of various genotypes demonstrate significant differences in both pathogenicity and tissue tropism (9). The earliest isolated strain, the Massachusetts (Mass) type, with M41 as the representative strain, predominantly infects the respiratory epithelial cells of chickens, leading to characteristic clinical symptoms such as dyspnea, sneezing, and tracheal rales (10). At present, the QX-type IBV is the most prevalent strain circulating worldwide. We have compared the classical strains like the Mass type M41 with prevalent QX-type IBV strains, and found the viruses exhibit notable differences in pathogenicity and antigenicity (11, 12). Among gamma-coronaviruses, it is noteworthy that all IBV strains possess an MBCS at the S1–S2 boundary, which constitutes a putative furin cleavage site (RXXR). Uniquely, the Beaudette strain also contains an additional furin cleavage motif (RRKR) at the S2′ site, which has been proved to be directly related to its Vero cell tropism (13, 14). The unique S2′ site has also been identified in Middle East respiratory syndrome coronavirus (MERS-CoV), conferring a special two-step furin activation for fusion on MERS-CoV (15). Our previous research has shown that incorporation of the Beaudette-derived furin-S2′ motif into YN leads to severe lesions in the central nervous system, with the mutant viruses exhibiting enhanced pathogenicity (16, 17). The replacement of the S protein, followed by the identification of three key amino acids surrounding the S2′ cleavage site, revealed the critical determinant for the Beaudette strain’s specific tropism and its ability to replicate in Vero cells (13). However, there still exist several critical questions: Is the S1/S2 cleavage site strictly essential for IBV replication? What specific biological function is conferred by the additional cleavage site at the S2′ position in the IBV strains? Furthermore, is there functional interplay between the S1/S2 and S2′ cleavage sites? Despite extensive research on the cleavage motif in other coronaviruses, the precise functional relationship between the conserved S1/S2 MBCS and the unique/engineered S2′ site in IBV pathogenesis and replication remains unclear, particularly across different genotypes in the context of chicken infection.

This study aims to elucidate the functional roles and potential synergistic relationship between the two cleavage sites at the S1/S2 and S2′ in relation to viral replication, tissue tropism, and pathogenicity. The QX-type IBV YN strain, which targets the urogenital system and the Massachusetts-type IBV M41 strain—which primarily infects the respiratory tract—were employed as model strains. Utilizing yeast transformation-associated recombination (TAR) technology, we efficiently generated recombinant viruses with various combinations of mutations at the S1/S2 and S2′ MBCSs. Our *in vitro* experiments highlighted the essential roles of the MBCSs at S1/S2 and S2′ in facilitating viral entry and cell–cell fusion. Furthermore, our *in vivo* challenge model provided direct evidence of the influence of the S1/S2 site on virulence and, for the first time, revealed that the function of the IBV S2′ cleavage site is contingent upon the presence of a multibasic motif at the S1/S2 site. This synergistic mechanism is conserved across IBV strains. These findings offer a theoretical foundation for the rational design of attenuated vaccines and effective control strategies against avian coronaviruses.

## RESULTS

### Rescue of recombinant viruses with mutations of the S1/S2 and S2′ cleavage site

**Fig 1:**
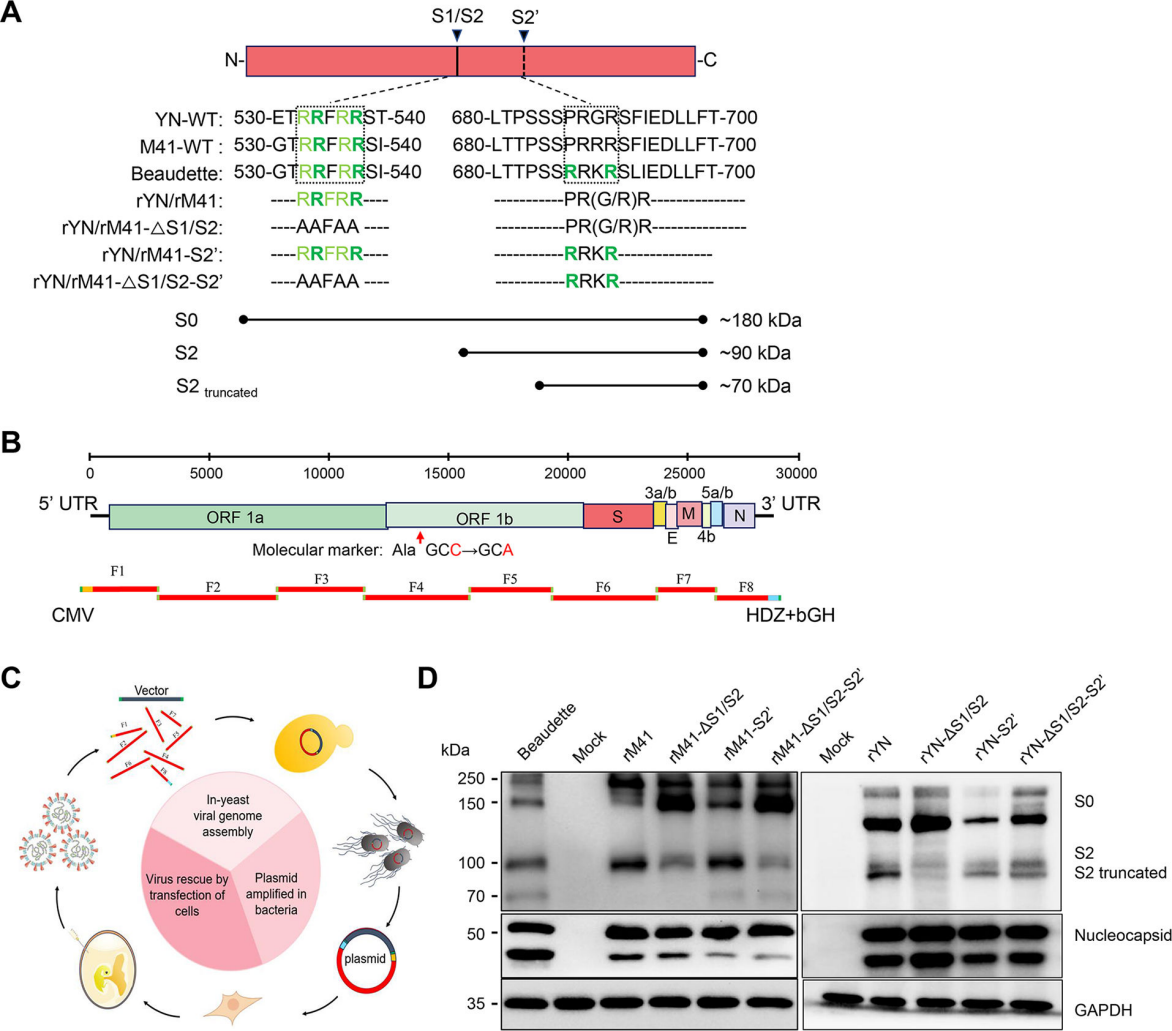
Construction and validation of recombinant IBV with mutations at cleavage sites. (**A**) Amino acid sequence alignment of the S1/S2 and S2' cleavage sites for the IBV strains and mutants used in this study. Key residues for protease recognition are highlighted in dark green. Mutants retaining potential cleavage sites are shaded in green, while those mutants with the sites ablated (ΔS1/S2) are shown in black; - indicates sequence identity. The schematic below the alignment illustrates the predicted molecular weights of the uncleaved full-length S glycoprotein (S0, ~180 kDa) and the S2 subunit (S2, ~90 kDa), including a truncated form (~70 kDa) based on the cleavage status at each site. (**B**) Schematic of the segmented viral genome assembly strategy. The full genome is split into eight fragments (F1–F8), each generated by PCR with 30–50 bp overlapping homology regions. The cassette consists of a CMV promoter, the 5′ untranslated region (UTR), the viral genome, the 3′ UTR, a hepatitis delta virus (HDV) ribozyme (Rz), and a bovine growth hormone (bGH) polyadenylation signal. A synonymous molecular marker (GCC→GCA, Ala) was introduced into the nsp12 gene (located in F4), with the mutated nucleotide shown in red. (**C**) General workflow for the rescue of recombinant IBV. The overlapping genomic fragments and a yeast shuttle vector were co-transformed into yeast for *in vivo* assembly via transformation-associated recombination (TAR). The assembled full-length plasmids were then electroporated into *E. coli* for amplification and sequencing. Validated plasmids were transfected into BHK-21 cells to initiate replication, and the resulting virus was subsequently propagated in SPF embryonated chicken eggs. (**D**) Western blot analysis of S protein cleavage. Lysates from CEK cells infected with the indicated viruses were probed with an anti-S2 monoclonal antibody. The positions of the full-length S0 glycoprotein and the S2 subunit are indicated. The blot demonstrates the cleavage phenotypes of the different mutants.

The sequence alignment of the spike protein revealed that the IBV YN, M41, and Beaudette possess a furin recognition motif (RRFRR) at the S1/S2 cleavage site. Notably, only the IBV Beaudette strain contains an additional MBCS (RRKR) at the S2′ position (Fig. 1A). As previously reported (18), we replaced arginine (R) with alanine (A) to disrupt the furin recognition motif, aiming to elucidate the specific functional roles at the S1/S2 and S2′ sites. Using TAR-based reverse genetics, we constructed panels of recombinant viruses based on both the YN and M41 genetic backbones. For each backbone, four distinct recombinant viruses were generated. (i) rYN/rM41: The parental virus contains the native MBCS at S1/S2 (RRFRR) and no S2′ site. (ii) rYN/rM41-ΔS1/S2 or rM41-ΔS1/S2: Viruses lacking MBCS at both S1/S2 and S2′ positions. The S1/S2 motif was mutated from RRFRR to AAFAA, and no modification was made at S2′. (iii) rYN-S2′**/**rM41-S2′: Viruses containing two MBCSs, retaining the native S1/S2 site (RRFRR) and introducing an engineered S2′ MBCS (RRKR). (iv) rYN-ΔS1/S2-S2′/rM41-ΔS1/S2-S2′: Viruses containing only one MBCS located at S2′. The S1/S2 MBCS was mutated from native RRFRR to AAFAA, while the furin recognition motif RRKR was introduced at the S2′ position. To recover recombinant viruses with distinct combinations of S1/S2 and S2′, the experimental design was achieved through a previously established strategy for generating recombinant IBV (19). The IBV genome was segmented into eight overlapping fragments, and a silent nucleotide substitution (C13880A) was introduced into the nsp12-encoding region to serve as a genetic marker, allowing differentiation between recombinant and wild-type viruses (Fig. 1B). The full-length genome was seamlessly assembled in *Saccharomyces cerevisiae* using transformation-associated recombination (TAR). Following the acquisition of the plasmid containing the complete IBV genome, BHK cells were transfected to recover infectious virus (Fig. 1C). The genetic stability of all eight recombinant IBV strains was verified through 10 serial passages in specific pathogen-free (SPF) embryonated chicken eggs (ECEs). RT-PCR amplification followed by Sanger sequencing of the targeted mutation sites verified that all engineered nucleotide substitutions (C13880A and cleavage site mutations) were stably maintained without reversion throughout the passaging process (data not shown).

The theoretical molecular weights of engineered furin-cleaved spike subunits were determined using the ExPASy ProtParam (Fig. 1A). The full-length S protein S0 has an estimated molecular weight of 180 kDa, while the cleaved S2 subunit is approximately 90 kDa. Additionally, the presence of an extra S2′ MBCS is predicted to yield a truncated S2 product of approximately 70 kDa. Western blot analysis confirmed protease-mediated proteolytic cleavage at the engineered mutation sites within the S protein. All rescued recombinant viruses, along with the Beaudette strain, were used to infect primary chicken embryonic kidney (CEK) cells. At 48 h post-infection (hpi), CEK cells were collected for Western blot analysis. The nucleocapsid (N) protein and the internal control GAPDH were expressed at comparable levels in whole-cell lysates from infected cells. Figure 1D revealed the presence of oligomers of S proteins (180–250 kDa), the uncleaved monomer form of the S glycoprotein (~180 kDa), and a cleaved form (S1/S2) of S2 at about 90 kDa. However, when the first MBCS at the S1/S2 boundary was disrupted in the mutants rM41/rYN-ΔS1/S2 and rM41/rYN-ΔS1/S2-S2′, the cleavage products of the S2 protein were markedly reduced compared to those in the parental rM41 and rYN. Notably, both rM41-S2′ and rM41-ΔS1/S2-S2′, which harbor an additional MBCS at the S2′ position, generated a truncated S2 product of approximately 70 kDa, comparable in size to that observed in Beaudette-infected cells.

### The S1/S2 cleavage site is critical for viral replication efficiency *in vitro*

**Fig 2:**
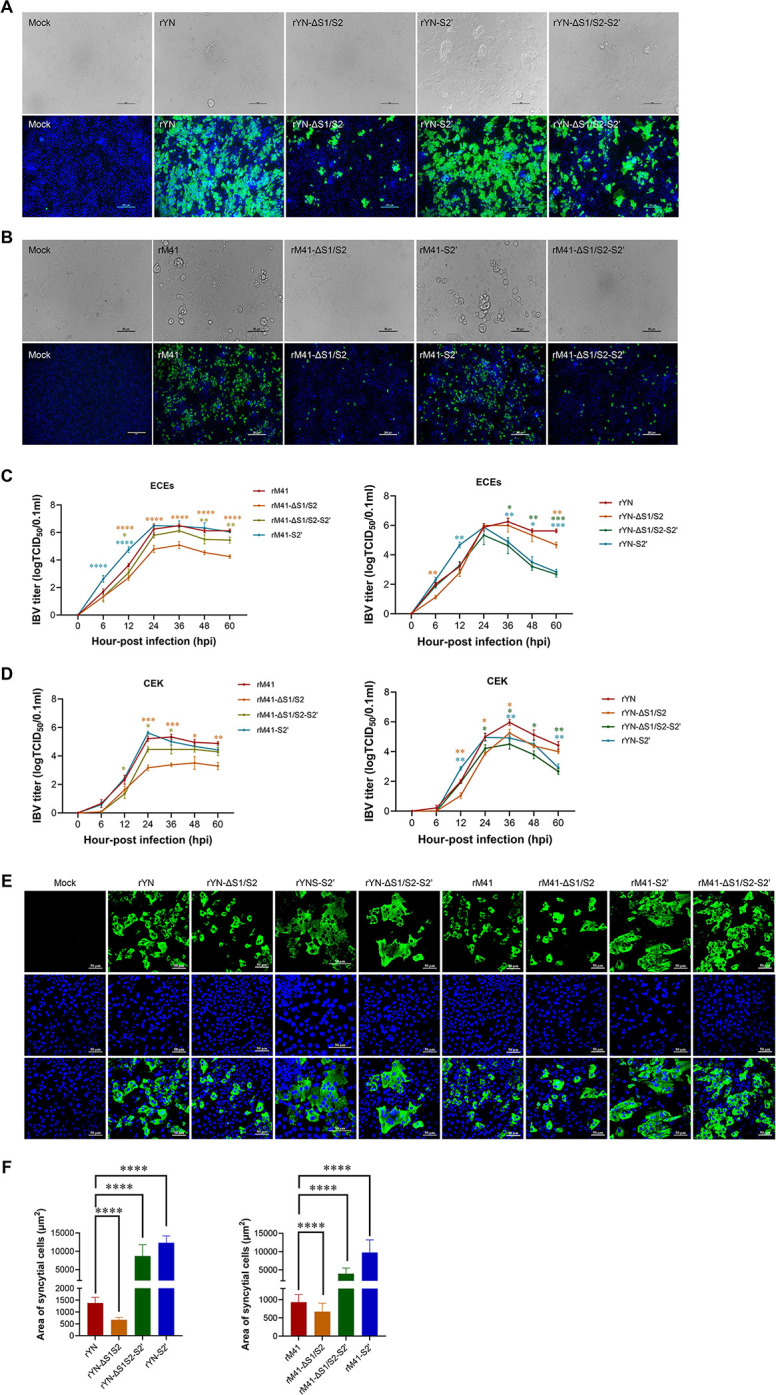
Growth capacity and membrane fusion activity of recombinant IBVs with cleavage site mutations *in vitro*. (**A and B**) Representative cytopathic effects (CPEs) induced by rYN-based (**A**) and rM41-based (**B**) viruses in CEK cells, as observed by bright-field (upper panels) and fluorescence microscopy (lower panels). Scale bar: 50 and 200 µm. (**C and D**) Replication kinetics of parental (rYN, rM41) and mutant viruses in embryonated chicken eggs (ECEs) (**C**) and chicken embryo kidney (CEK) cells (**D**). Allantoic fluid or cell culture supernatant was collected at 6, 12, 24, 36, 48, and 60 hpi, and viral titers were measured in CEK cells. Data are presented as mean log_10_ TCID_50_ ± SD (*n* = 3). Statistical significance was determined by two-way ANOVA; *P < 0.05, **P < 0.01, ***P < 0.001, and ****P < 0.0001. (**E**) Syncytium formation in CEK cells infected with the indicated viruses at 16 hpi. Cells were immunostained for the viral nucleocapsid (N) protein (green). Nuclei were counterstained with DAPI (blue). Images were acquired by confocal microscopy. Scale bar: 50 µm. (**F**) Quantitative analysis of cell-cell fusion. The syncytial area from panel E was measured using ImageJ software. Data are presented as mean ± SD (*n* = 8). Statistical significance was determined by unpaired *t*-test; *P < 0.05, **P < 0.01, ***P < 0.001, and ****P < 0.0001.

For comparison of the growth properties of rescued viruses, the cytopathic changes of CEK cells were observed after infection with wild-type IBVs and their cleavage site-modified counterparts (Fig. 2A and B). The parental strains rYN and rM41 induced extensive CPE and large areas of immunofluorescence, indicative of robust infection. In contrast, deletion of the S1/S2 MBCS (rYN/rM41-ΔS1/S2) markedly reduced both the number of antigen-positive cells and detectable CPE. The introduction of an engineered S2' MBCS in the double mutant (rYN/rM41-ΔS1/S2-S2′) partially restored infectivity, with more evident CPE than the S1/S2-deleted mutant, though not to wild-type levels. Strikingly, mutants carrying the S2' cleavage site (rYN/rM41-S2′) induced the most severe CPE, characterized by pronounced syncytium formation, cell rounding, and detachment-indicative of enhanced cell-to-cell spread (Fig. 2A and B).

The multi-step growth kinetics of the viruses in both ECEs and CEK cells were also determined by observation of cytopathic effect, and the TCID_50_ at each time point were calculated according to Reed and Muench (Fig. 2C and D). In ECEs and CEK cells, deletion of the S1/S2 site severely impaired viral replication of rM41. This defect was partially restored by introducing the S2′ site, with the rM41-ΔS1/S2-S2′ displaying intermediate titers between the S1/S2-deleted mutant and the wild-type rM41. The rM41-S2′ mutant showed accelerated early growth, but declined after 24 hpi in CEK cells. For rYN, the S1/S2 deletion also reduced replication, but the effect was less pronounced: in ECEs, rYN-ΔS1/S2 reached a peak similar to wild type at 24 hpi but declined faster at later time points. In CEK cells, the S1/S2 deletion (rYN-ΔS1/S2) reduced titers throughout. Interestingly, the rYN-ΔS1/S2-S2′ and the rYN-S2′ both exhibited rapid early replication but then dropped sharply after 24 hpi, much lower than wild-type rYN. These results indicate that while the S1/S2 site is critical for efficient replication in both ECE and CEK, the CEK environment amplifies the deleterious effects of S2′ introduction, especially in the rYN background, which may reflect the differential S2′ cleavage dependence of the two IBV serotypes in CEK cells (Fig. 2D).

To quantitatively assess membrane fusion activity, we measured syncytia area in infected CEK cells via immunostaining and image analysis (Fig. 2E and F). Loss of the S1/S2 MBCS significantly reduced the area of fluorescence-positive syncytia compared to parental viruses. Conversely, the presence of the S2′ MBCS substantially increased syncytia size, confirming its role in promoting cell-cell fusion. These results collectively indicate that the S1/S2 MBCS is critical for efficient IBV replication *in vitro*. Its deletion severely attenuates viral growth and spread. However, the introduction of a cleavage site at S2′ partially compensates for this defect by enhancing syncytia formation and restoring limited replication capacity.

### Loss of the S1/S2 MBCS results in reduced virulence *in vivo*, whereas the presence of S2′ MBCS leads to exacerbated symptoms

**Fig 3:**
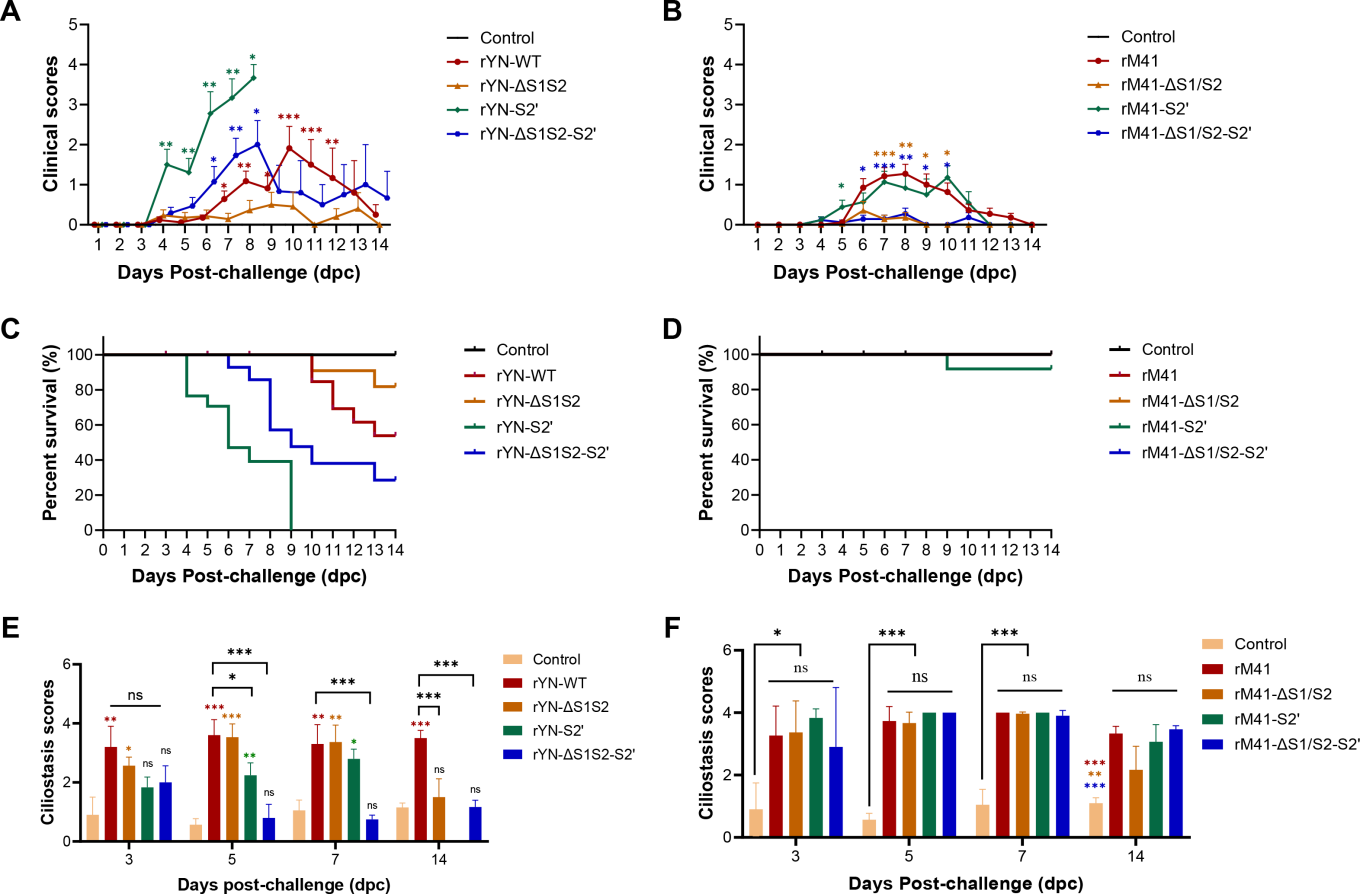
Pathogenicity of recombinant IBVs with cleavage site mutations *in vivo*. One-day-old SPF chickens infected with the indicated parental or mutant viruses. (**A and B**) Daily clinical scores of chickens infected with rYN-derived (**A**) or rM41-derived (**B**) viruses. Clinical signs were scored on a scale of 0–4: 0, healthy; 1, mild depression, rales, watery droppings, slight head shaking, hunched posture, or lacrimation; 2, sneezing, coughing, marked depression, watery droppings, or nasal discharge; 3, open-mouth breathing, neurological symptoms, or severe watery droppings; and 4, moribund or dead. (**C and D**) Survival curves of chickens infected with rYN-derived (**C**) or rM41-derived (**D**) viruses. (**E and F**) Ciliostasis scores in the tracheas of chickens infected with rYN-derived (**E**) or rM41-derived (**F**) viruses at 3, 5, 7, and 14 dpc. Ciliary activity was assessed based on the following criteria: 0 = 100% ciliary activity; 1 = 75–100% ciliary activity; 2 = 50–75% ciliary activity; 3 = 25–50% ciliary activity; and 4 = 0–25% ciliary activity. Data are presented as mean ± SD. Statistical significance was determined by two-way ANOVA for clinical and ciliostasis scores and Log-rank test for survival curves. *P < 0.05, **P < 0.01, and ***P < 0.001.

To assess the pathogenicity of cleavage site-modified IBVs *in vivo*, 1-day-old SPF chickens were intranasally inoculated with wild-type rYN and rM41 viruses or their respective mutants. Clinical signs were monitored and scored daily post-challenge (dpc). Initial symptoms, including depression, rales, ruffled feathers, watery diarrhea, coughing, open-mouth breathing, and occasional mortality, emerged at 4 dpc. Consistent with known serotype-specific pathogenicity reported in previous studies (20), chickens infected with rYN-derived mutants exhibited substantially more severe clinical signs (Fig. 3A) than those infected with rM41-based strains (Fig. 3B). Notably, the presence of the S2′ MBCS was strongly associated with enhanced virulence. Viruses carrying the site (rYN-S2′ and rM41-S2′) induced the most severe clinical manifestations, with peak severity occurring between 6 and 8 dpc. In the rYN-S2′ group, all birds developed pronounced neurological signs and succumbed to infection by 8 dpc (Fig. 3C). The rM41-S2′ group also showed heightened pathogenicity, resulting in one mortality at 9 dpc (Fig. 3D). In contrast, deletion of the S1/S2 MBCS significantly attenuated both rYN-ΔS1/S2 and rM41-ΔS1/S2, which induced only mild clinical signs and reduced mortality. The double mutants (rYN-ΔS1/S2-S2′ and rM41-ΔS1/S2-S2′) exhibited intermediate phenotypes. In the rYN background, the introduction of S2′ partially restored virulence: 7 of 10 chickens in the rYN-ΔS1/S2-S2′ group developed neurological signs and died within 14 dpc. By contrast, rM41-ΔS1/S2-S2′ remained attenuated, similar to the rM41-ΔS1/S2 group, with only transient tracheal rales and no mortality, highlighting the influence of the viral backbone on the pathogenic outcome. Tracheal ciliostasis scores further underscored these strain-dependent differences (Fig. 3E and F). The respiratory-tropic rM41 strain caused severe ciliary damage regardless of cleavage status, with complete ciliostasis observed by 7 dpc across all groups and partial recovery by 14 dpc (Fig. 3F). In contrast, rYN variants carrying the S2′ MBCS (rYN-S2′ and rYN-ΔS1/S2-S2′) exhibited significantly reduced tracheal damage at 5, 7, and 14 dpc (Fig. 3E), suggesting that enhanced neurotropism mediated by the S2′ site may come at the expense of respiratory tropism. Notably, the impact of cleavage site mutations was less pronounced in the rM41 background compared to the rYN strain, suggesting that the M41 genomic backbone is inherently less dependent on furin-mediated cleavage for pathogenicity in this model.

### The S1/S2 cleavage site governs the original tissue pathogenicity, while the S2′ site amplifies neurological organ-specific damage

**Fig 4:**
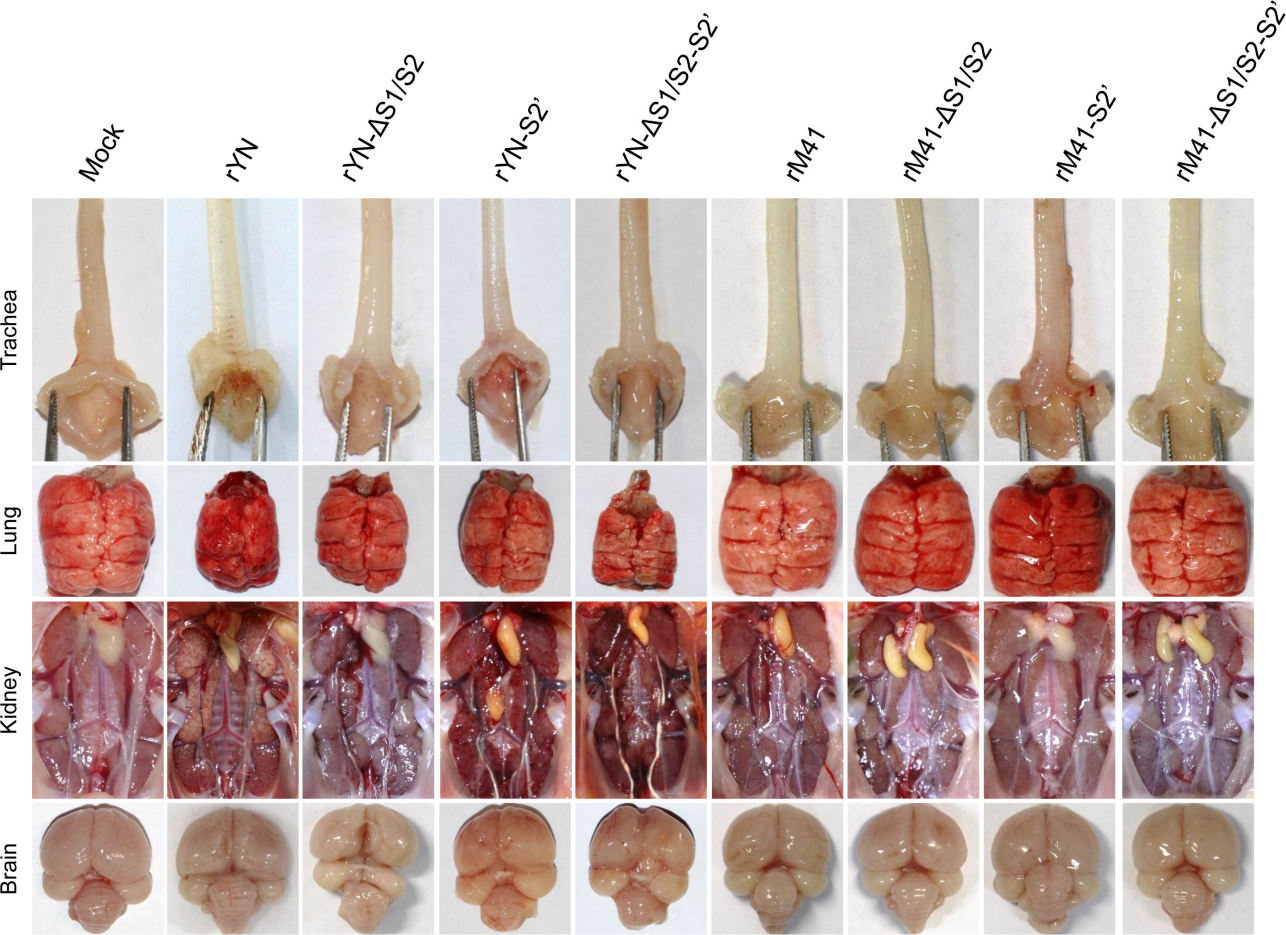
Gross lesions induced by recombinant IBVs with distinct cleavage site combinations. Representative images of gross pathological changes in the trachea, lung, kidney, and brain of 1-day-old SPF chickens at 5 days post-infection with the indicated viruses. The Mock group represents uninfected control chickens.

Pathological examination at 5 dpc further elucidated the tissue tropism and organ-specific pathogenicity of the cleavage site-modified viruses (Fig. 4). In chickens infected with the wild-type rYN strain, severe lesions—including severe hemorrhage and urate deposition—were observed in the trachea, lungs, and kidneys. In contrast, both rYN-ΔS1/S2 and rYN-ΔS1/S2-S2′ induced markedly attenuated pathology in these organs, underscoring the critical role of the native S1/S2 cleavage site in original tissue pathogenicity. Notably, the mutant IBVs with the S2′ MBCS, rYN-ΔS1/S2-S2′ and rYN-S2′, caused enhanced damage in the brain, indicating a shift toward neurotropism. In line with its known respiratory tropism, rM41 and its derivative mutants primarily induced pathology in the respiratory tract, with no remarkable lesions detected in the kidneys or brain. Tracheal hemorrhage was observed in all birds infected with rM41 and rM41-S2′ (3/3), whereas only one of three chickens in the rM41-ΔS1/S2 and rM41-ΔS1/S2-S2′ groups exhibited mild laryngeal petechial hemorrhage. Additionally, lungs from the rM41-S2′ group appeared slightly enlarged and were covered with viscous hemorrhagic exudate.

**Fig 5:**
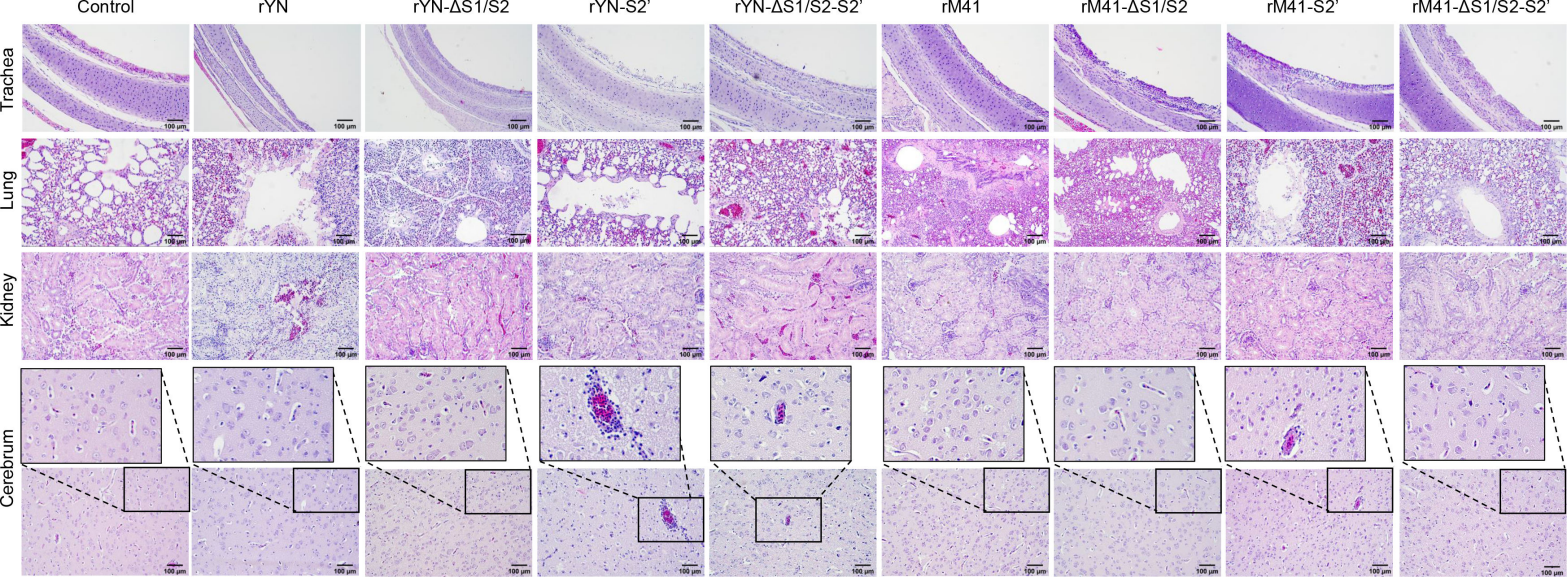
Histopathological analysis of tissue lesions induced by recombinant IBVs with distinct cleavage sites. Tissue sections (trachea, kidney, lung, and cerebrum) were collected from 1-day-old SPF chickens at 5 days post-infection and stained with hematoxylin and eosin (H&E). Scale bar = 100 µm (magnification, ×200). Representative images from each group are shown. Key pathological features observed include the following: trachea—epithelial desquamation, lymphocyte infiltration, and edema; kidney—interstitial nephritis, tubular degeneration, and urate deposition; lung—pneumonia, hemorrhage, and inflammatory cell infiltration; and cerebrum—gliosis, perivascular cuffing, and neuronal degeneration. The Mock group represents uninfected controls.

Histopathological examination of primary target organs (trachea, lung, and kidney) and the cerebrum was performed at 5 dpc to further characterize tissue-specific lesions induced by the cleavage site-modified viruses (Fig. 5). Consistent with gross pathology findings, all infected groups exhibited notable damage to the trachea, including epithelial desquamation, thickening, degeneration, necrosis, and loss of tracheal epithelial cilia. In the lungs, infection with rYN and rM41 strains resulted in hemorrhage, congestion, and accumulation of erythrocytes and lymphocytes within bronchial and air capillary lumina. Renal injury was particularly severe in rYN-infected chickens, showing marked tubular epithelial degeneration and necrosis, intraluminal cellular debris, and extensive lymphoplasmacytic interstitial infiltration. In contrast, both rYN-ΔS1/S2 and rM41-ΔS1/S2 mutants induced only mild inflammatory and pathological changes in the lung and kidney, confirming that deletion of the S1/S2 site significantly attenuates tissue damage in primary target organs. Notably, cerebral lesions—characterized by perivascular cuffing and hyperemia—were exclusively observed in chickens infected with the mutant IBVs carrying the S2′ MBCS (rYN-ΔS1/S2-S2′ and rYN-S2′), indicating that the introduced S2′ MBCS confers neurotropic potential. Collectively, these results demonstrate that the deletion of S1/S2 MBCS is essential for full pathogenicity in respiratory and renal tissues, while engineering of the S2′ cleavage site shifts virulence toward the central nervous system.

### The S1/S2 and S2′ cleavage sites drive pathogenic divergence via altered tissue tropism

**Fig 6:**
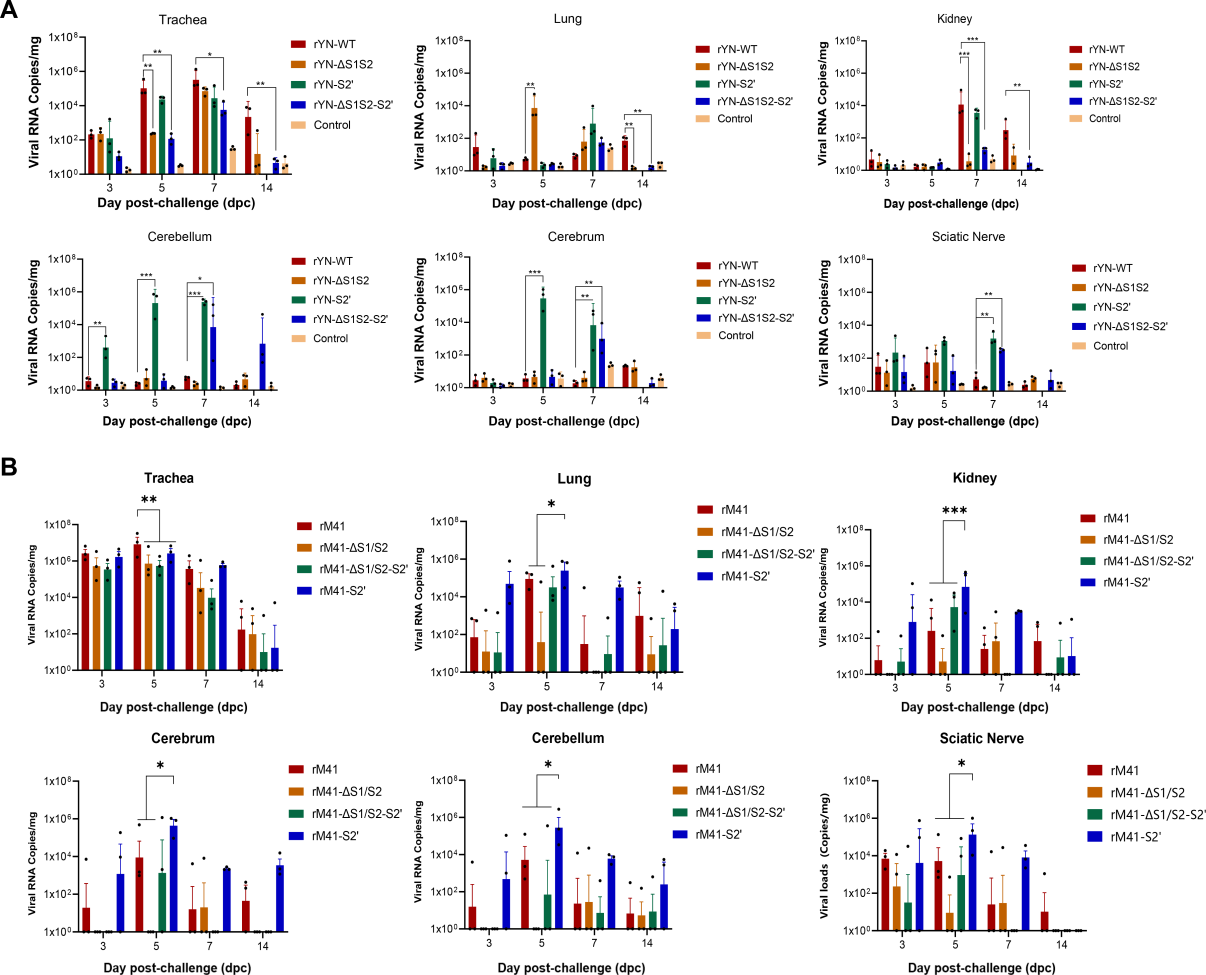
Tissue viral loads in chickens infected with recombinant IBVs with different combinations of cleavage sites. Viral loads in the trachea, lung, kidney, cerebrum, cerebellum, and sciatic nerve of 1-day-old SPF chickens at 3, 5, 7, and 14 days post-infection were quantified by qRT-PCR. (**A**) Viral loads in tissues from chickens infected with rYN-derived viruses. (**B**) Viral loads in tissues from chickens infected with rM41-derived viruses. Data are presented as mean log_10_ copies per milligram of tissue ± SD. Statistical significance was determined by two-way ANOVA relative to the respective parental strain (rYN or rM41) at each time point. *P < 0.05, **P < 0.01, and ***P < 0.001.

Given the marked differences in pathogenicity among the mutants, we sought to determine whether these phenotypes were correlated with alterations in organ-specific replication. We quantified viral loads in the trachea, lung, kidney, cerebrum, cerebellum, and sciatic nerve of infected chickens by qRT-PCR. As shown in Fig. 6A, both wild-type and mutant YN strains replicated in the trachea, although viruses lacking the S1/S2 MBCS exhibited delayed peak kinetics. In the lung, the rYN-ΔS1/S2 mutant showed significantly increased viral loads at 5 dpc. Renal replication was most prominent in the rYN and rYN-S2′ groups, which displayed significantly higher titers than other mutants at 7 dpc. Notably, neurotropic replication was strongly associated with the S2′ MBCS. The rYN-S2′ virus was detected in the cerebellum as early as 3 dpc, with peak titers at 5–7 dpc—temporally coinciding with the mortality observed in this group. Although rYN-ΔS1/S2-S2′ also reached high viral loads in the cerebrum and cerebellum, its replication in neural tissues was delayed compared to rYN-S2′. In chickens infected with rM41-derived viruses (Fig. 6B), high viral loads were consistently detected in the trachea from 3 to 7 dpc across all groups. Replication in other tissues peaked transiently at 5 dpc before rapidly declining. Notably, rM41-S2′ achieved significantly higher viral loads in the kidney, cerebellum, and sciatic nerve at 5 dpc compared to the other three rM41-based viruses. In the lung and cerebrum, both rM41-S2′ and rM41-ΔS1/S2-S2′ showed comparable replication levels, which were significantly elevated relative to rM41 and rM41-ΔS1/S2.

**Fig 7:**
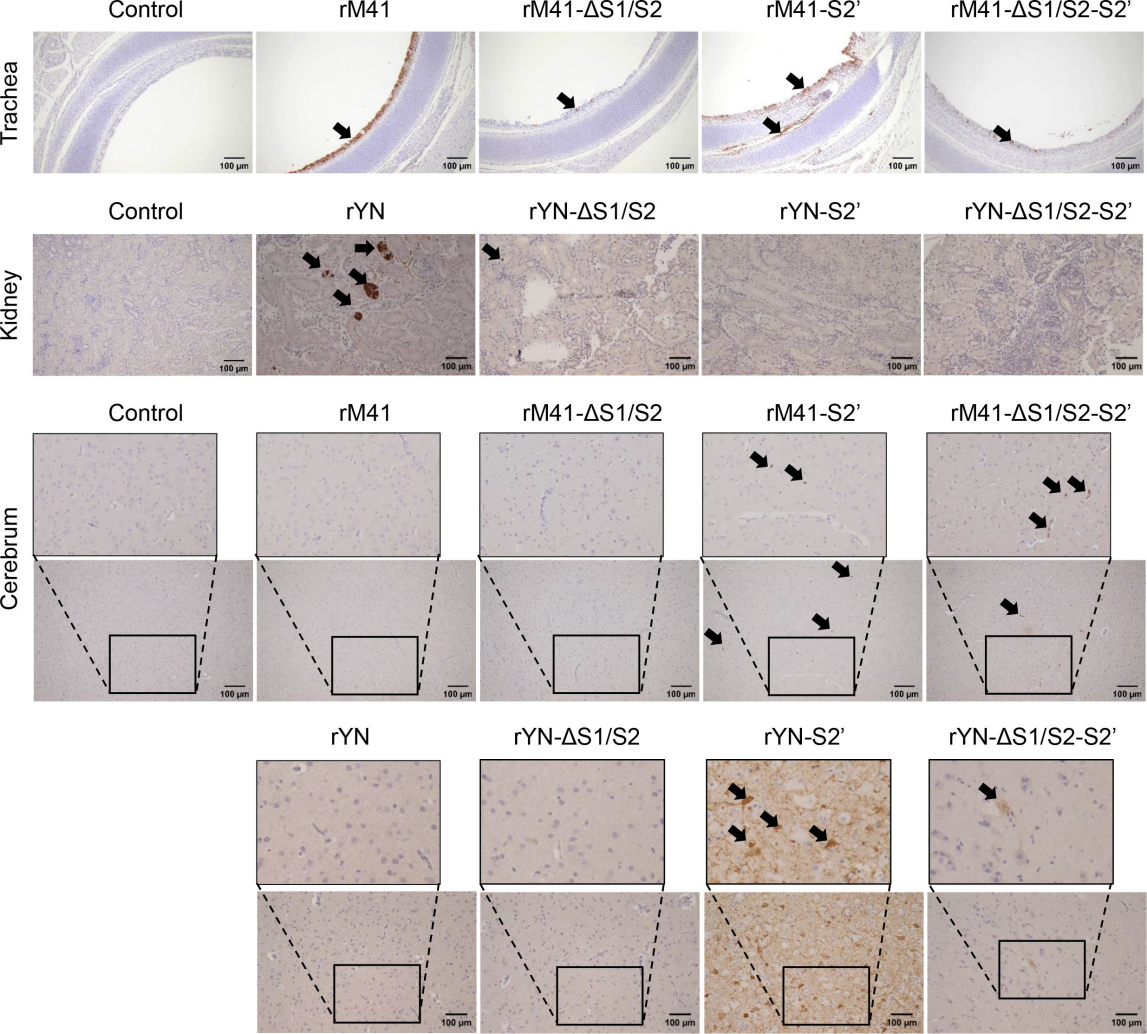
Immunohistochemical detection of IBV antigen in tissues of infected SPF chickens. Tissue sections from the trachea, kidney, and cerebrum were collected at 5 days post-infection from 1-day-old SPF chickens infected with the indicated rYN- or rM41-derived viruses. Sections were immunostained using a monoclonal antibody against the IBV nucleocapsid (N) protein, followed by DAB development (brown) and hematoxylin counterstaining (blue). IBV N protein-positive cells are indicated by black arrows. Scale bar, 100 µm.

Immunohistochemical (IHC) analysis further corroborated the organ-specific replication patterns of the mutants, revealing clear differences in antigen distribution across tissues (Fig. 7). In the M41-based virus-infected groups, IBV antigen-positive cells were consistently observed in the tracheal epithelium across all infected groups. However, there was a notable reduction in the number of infected cells in the trachea among the S1/S2 cleavage site deletion mutants (rM41-ΔS1/S2 and rM41-ΔS1/S2-S2′), which aligns with the viral loads measured by quantitative reverse transcription-PCR (RT-qPCR). In the kidney, strong IBV N protein signals were exclusively detected in the rYN-infected group, while all mutants showed markedly weaker staining, indicating that disruption of the native cleavage sites attenuates renal tropism. Notably, the introduction of the S2′ MBCS was associated with neuroinvasion. Viral antigen was clearly visualized in the cerebrum of chickens infected with S2′-carrying mutants, including rYN-S2′, rYN-ΔS1/S2-S2′, and their rM41-derived counterparts. Although rM41-S2′ and rM41-ΔS1/S2-S2′ exhibited only focal positive staining, rYN-S2′ infection resulted in extensive N protein deposition throughout the brain parenchyma, consistent with its severe neuropathogenicity. The IHC findings demonstrate that the S1/S2 MBCS is critical for efficient replication in primary target organs such as the trachea and kidney, while the engineered S2′ site enables viral spread to the central nervous system. Moreover, the presence of the S1/S2 cleavage site further facilitates the S2′-mediated enhancement of IBV tissue tropism, which drives pathogenic divergence of different IBV strains.

### Host protease utilization is determined by cleavage site composition and viral backbone

**Fig 8:**
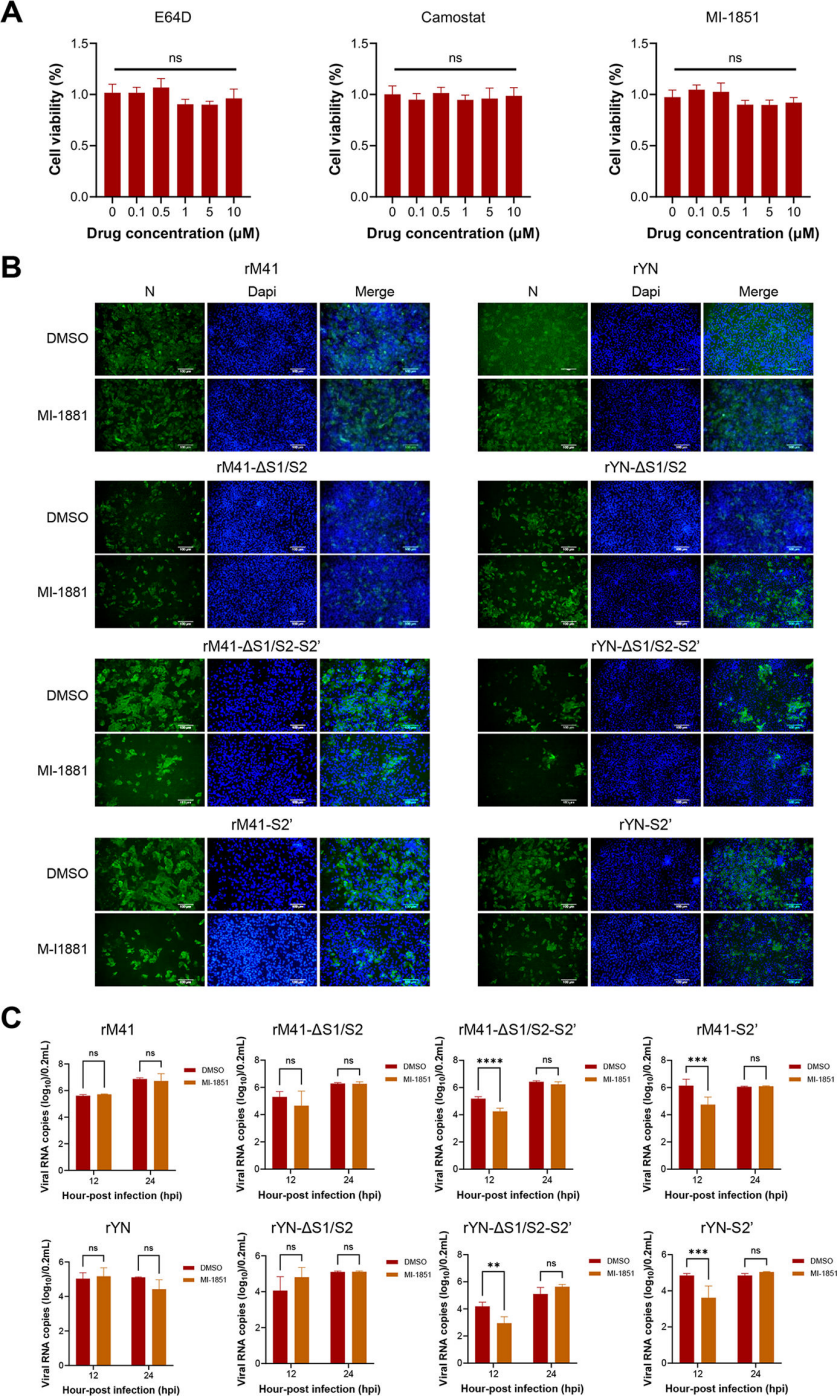
Impact of furin inhibition on IBV infectivity *in vitro*. (**A**) Effects of host protease inhibitors on cell viability. Cell viability of CEK cells was measured by the CCK-8 assay after 24 h of incubation with increasing concentrations (0–10 µM) of E64D, camostat, and MI-1851. All three inhibitors did not affect the cell viability up to 10 μM concentration (*n*  =  3; *P* > 0.05). (**B and C**) CEK cells were pretreated with MI-1851 in a concentration of 10 µM or DMSO control for 2 h, then infected with the indicated IBV strains. Immunofluorescence analysis of the expression of IBV nucleoprotein after infection was conducted at 12 hpi. Green indicates staining of IBV N protein. Blue indicates cell nucleus stained with DAPI (**B**), and viral RNA copy number in the supernatant and cell lysates was quantified by RT-qPCR at 12 and 24 hpi (**C**). Data are presented as mean Log_10_ viral RNA copy number/0.2 mL ± standard deviation (*n* = 3). Statistical analysis: two-way ANOVA (**P < 0.01, ***P < 0.001, and ****P < 0.0001). ns, not significant (*P* ≥ 0.05).

To define the mechanism of S protein cleavage and its functional impact on viral replication, we assessed the roles of different host proteases using specific inhibitors. The effects of MI-1851, E64D, and camostat on cell viability were first measured by the CCK-8 assay (Fig. 8A). The viability of CEK cells remained above 90% across all inhibitor treatment conditions. Therefore, we chose the maximum safe concentration (10 µM) for subsequent experiments. Furin is a well-recognized protease that processes MBCS. Accordingly, we first evaluated the antiviral effect of the specific furin inhibitor MI-1851 on the replication of different IBV strains. Interestingly, MI-1851 significantly suppressed the replication of mutants carrying the S2′ site (rM41/rYN-ΔS1/S2-S2′ and rM41/rYN-S2′), with the inhibitory effect primarily observed at 12 hpi, but had little effect on wild-type viruses or S1/S2-deleted mutants (Fig. 8B and C). These results suggest that cleavage at the S1/S2 site may not be primarily mediated by furin or that alternative proteases can compensate for furin deficiency.

**Fig 9:**
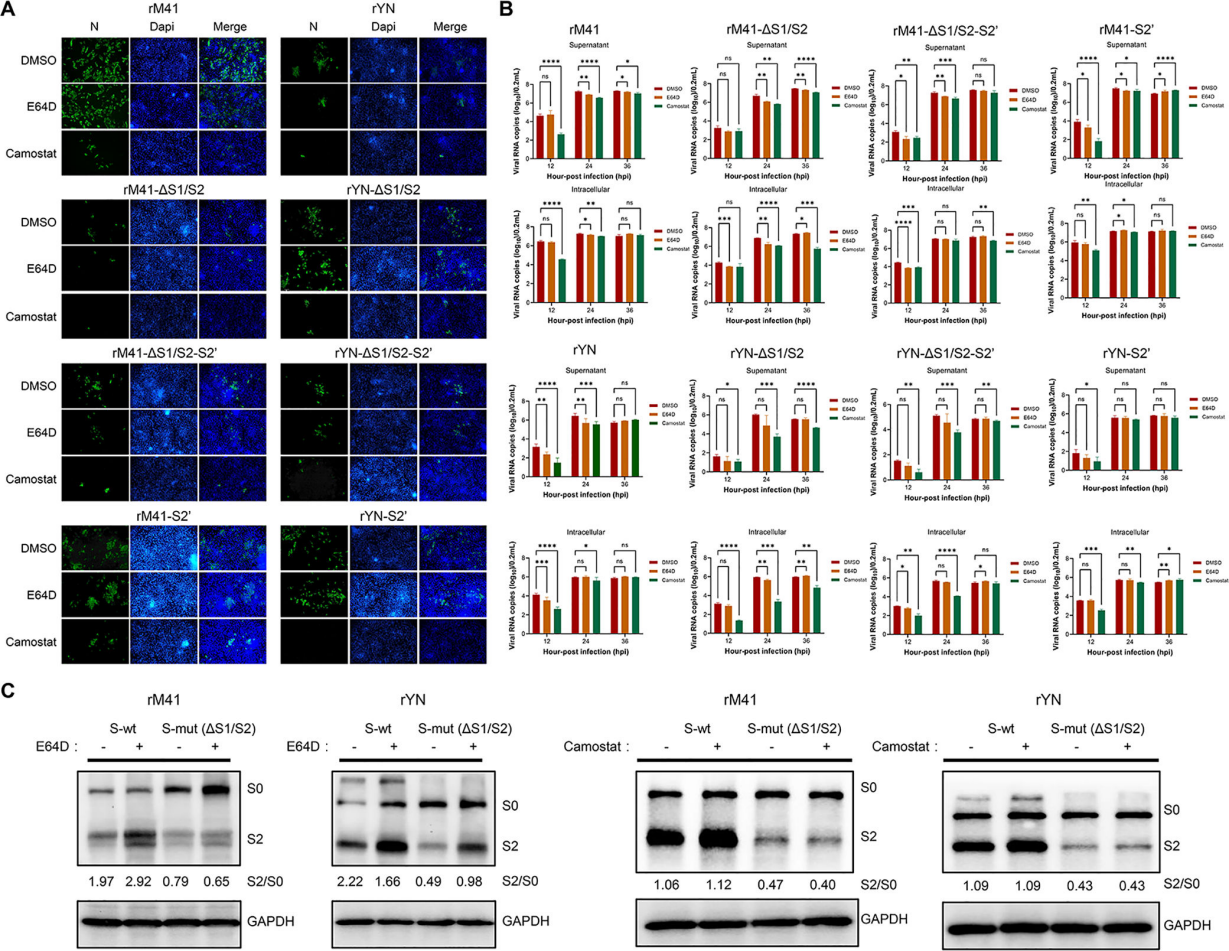
Effects of the TMPRSS2 inhibitor camostat and the cathepsin L inhibitor E64D on IBV infectivity and S protein cleavage patterns. CEK cells were pretreated with camostat, E64D in a concentration of 10 µM or DMSO control for 2 h, then infected with the indicated IBV strains. Immunofluorescence analysis of the expression of IBV nucleoprotein after infection was conducted at 12 hpi. Green indicates staining of IBV N protein. Blue indicates cell nucleus stained with DAPI (**A**), and viral RNA copy number in the supernatant and cell lysates was quantified by RT-qPCR at 12, 24, and 36 hpi (**B**). Data are presented as mean Log_10_ viral RNA copy number/0.2 mL ± standard deviation (*n* = 3). Statistical analysis: two-way ANOVA (*P < 0.05, **P < 0.01, ***P < 0.001, and ****P < 0.0001). ns, not significant (*P* ≥ 0.05). (**C**) Western blot analysis of rM41 and rYN S protein cleavage in the presence of E64D or camostat. HEK 293T cells were pretreated with 10 µM E64D or camostat for 2 h, then transfected with spike constructs and cultured for 24 h. Cleavage products were detected using a mouse monoclonal antibody against the S2 protein (prepared in-house, diluted 1:5,000 in PBS) and an anti-GAPDH antibody as an internal control. S0: full-length S protein, S2: cleaved S2 subunit. The S2/S0 ratio beneath each lane indicates cleavage efficiency, as determined by densitometric analysis using ImageJ software.

To further investigate this, we next examined two other host proteases known to be involved in coronavirus entry: the serine protease TMPRSS2 and the cysteine protease cathepsin L (CTSL), using their specific inhibitors camostat and E64D, respectively (Fig. 9A and B). Both wild-type strains relied heavily on the serine protease pathway, as camostat treatment dramatically reduced viral replication in both groups. The inhibitory effect of camostat was more pronounced in the absence of the S1/S2 cleavage site. However, the two strains differed in their dependence on the CTSL-mediated endosomal pathway: rM41 showed minimal dependence on the cathepsin-mediated endosomal pathway, with E64D treatment having no effect on its early replication at 12 hpi. In contrast, rYN can also utilize the cathepsin-mediated pathway. E64D treatment reduced its viral loads significantly at 12 hpi. Moreover, cleavage site modifications significantly altered protease dependence. Mutation of the S1/S2 site in YN substantially reduced its reliance on CTSL. We also noticed that the inhibitory effects of both camostat and E64D on viral replication gradually diminished at later time points (especially 36 hpi).

The effects of E64D and camostat on S protein processing were further assessed by Western blot analysis (Fig. 9C). Under E64D treatment, the S1/S2 cleavage patterns differed among the recombinant viruses. In rM41 and rYN-ΔS1/S2, E64D did not reduce the S1/S2 cleavage-associated band pattern and in some experiments, appeared to increase the relative S1/S2 signal. In contrast, rYN and rM41-ΔS1/S2 showed an apparent reduction in the S1/S2-associated band ratio after E64D treatment. Camostat treatment did not significantly alter the S2/S0 ratio in any of the tested viruses. Although these Western blot data do not allow definitive assignment of the specific protease(s) involved in S processing, the distinct inhibitor-associated banding patterns observed among the recombinant viruses are consistent with differential protease responsiveness. When considered together with the inhibitor-based viral RNA quantification and IFA results, these findings further support the notion that alterations in the cleavage-site context can modulate protease dependence during IBV entry.

## DISCUSSION

The cleavability of viral envelope glycoproteins is a critical prerequisite for the infectivity and pathogenicity of enveloped viruses. Proteolytic activation of viral fusion proteins is a crucial determinant of host adaptation and pathogenicity across various viruses, including HIV-1, human parainfluenza virus type 3 (HPIV3), Ebola virus, and avian influenza virus (21). For example, the fusion protein (F) of HPIV3 requires proteolytic cleavage by host proteases to become fusion-competent, enabling viral entry (22). Similarly, the S protein of coronaviruses serves as the primary determinant for viral entry, as it possesses receptor-binding and fusion functions (23). While binding to host cell receptors constitutes the essential first step in establishing infection, the protease activation phase is critical for the spike protein’s fusion capability. In many coronaviruses, cleavage at the S1/S2 boundary occurs during biosynthesis, yielding a mature virion with two non-covalently linked subunits (S1 and S2) (24). The S1/S2 cleavage is a prerequisite for subsequent cleavage of the S2 cleavage site by host protease, exposing the fusion peptide to fuse with the host cell membrane (25).

Different CoVs have evolved multiple ways to achieve proteolytic priming. Most of the known coronaviruses (MERS-CoV, HCoV-OC43, HCoV-HKU1, SARS-CoV-2, and IBV) contain a multibasic cleavage site at the S1/S2 junction site, which has been proven experimentally to confer cleavage by proprotein convertases like furin (26). Only a few other coronaviruses are known for having different amino acid sequence motifs of the proteolytic cleavage site. For instance, the SARS-CoV and bat SARS-like coronaviruses lack this multibasic site and are not cleaved by furin at the S1/S2 boundary (27). Similarly, the seasonal human coronaviruses HCoV-NL63 and HCoV-229E do not contain a multibasic furin cleavage site at the S1/S2 junction; instead, they rely on dibasic or monobasic arginine residues for entry, and their fusion activation appears to be a one-step process that does not require prior S1/S2 cleavage (28). The S2 protein has another proteolytic site (S2′) that lies immediately upstream of the S2 subunit fusion peptide (FP) domain, which is also important for the virus entry process. Although the precise nucleotide sequences of the S2′ site vary among different coronaviruses, the encoded amino acid motif generally features multiple arginine (R) and lysine (K) residues that are recognized by host proteases such as furin, TMPRSS2, or cathepsins, which are essential for efficient cleavage.

The presence of an MBCS in the spike protein is generally considered to enhance viral entry and infectivity. In SARS-CoV-2, the S1/S2 MBCS (682–RRAR–685) has been shown to enhance both infectivity and cell-cell fusion ability (5, 29, 30). Accordingly, deletion of the S1/S2 MBCS attenuates SARS-CoV-2 pathogenicity in hamster and ferret models (31, 32), underscoring its crucial role in virulence. In IBV, an additional MBCS (RRKR) at the S2′ site confers expanded mammalian cell tropism to the Vero cell-adapted Beaudette strain (13). This strain was derived through serial passage in chicken embryos and Vero cells, during which mutations that enhance replication in cultured cells were selectively enriched (33). In contrast, most epidemic IBV strains replicate poorly or not at all in Vero cells. Our previous work demonstrated that introducing an MBCS (RRKR) into the S2′ site of the virulent YN strain significantly enhanced pathogenicity, causing increased mortality and severe neurological symptoms in SPF chickens, with confirmed neuroinvasion and encephalitis (17). Notably, the role of MBCS in the S protein function is not universally conserved. For example, among feline coronaviruses, isolates from healthy cats often possess an optimized furin cleavage sequence (RRARR↓S) at the S1/S2 boundary, whereas this sequence is frequently mutated or lost in feline infectious peritonitis virus (FIPV) strains associated with systemic disease. This observation suggests that loss of the MBCS may correlate with altered pathogenesis, highlighting the context-dependent nature of these cleavage motifs (34).

It remains unclear whether the S1/S2 cleavage site is indispensable for IBV replication, what functional role an introduced S2′ site might play across different IBV strains, and whether potential interactions exist between the S1/S2 and S2′ cleavage sites. To address these questions, we successfully rescued recombinant IBVs (rYN and rM41 backbones) with mutations in the S1/S2 and S2′ cleavage sites (Fig. 1). The successful rescue of viruses lacking the conserved S1/S2 MBCS (rYN/rM41-ΔS1/S2 and rYN/rM41-ΔS1/S2-S2′) demonstrates that this cleavage site is not absolutely essential for IBV viability. However, these S1/S2 cleavage-deficient mutants exhibited significantly impaired replication efficiency in both ECEs and CEK cells (Fig. 2C and D). Specifically, in ECEs, deletion of the S1/S2 site severely impaired rM41 replication, whereas the effect on rYN was less pronounced, with rYN-ΔS1/S2 reaching a peak similar to wild-type but declining faster at later time points. These results indicate that while the S1/S2 cleavage sites are not strictly indispensable for establishing infection, they play a crucial role in optimizing viral replication. These findings are in agreement with previous observations in SARS-CoV-2, where deletion of the S1/S2 MBCS reduced replication in human lung cells but did not abolish viability (5). Introducing the S2′ cleavage site into the S1/S2-deficient mutant rM41-ΔS1/S2-S2′ partially restored replication levels, though not to wild-type levels. This partial recovery suggests that the S2' cleavage site may enhance early cell-to-cell spread, consistent with a previous report that efficient cleavage at the S2′ site by TMPRSS2 could strongly attenuate or abolish the infectivity difference between wild-type and MBCS mutants and rescue syncytium formation (35). Supporting this, our CPE and IFA results revealed restored infectivity (Fig. 2A and B) as well as enhanced syncytium formation (Fig. 2E and F) in CEK cells infected with mutants carrying the S2′ cleavage site compared to the parental strains and the S1/S2-deficient mutants.

Notably, the extent of replication restoration by the S2′ site was dependent on both the viral genetic background and the host cell type. The rM41 backbone appeared more permissive for S2′-mediated compensation, particularly in ECEs, whereas the rYN backbone exhibited more severe attenuation that was only modestly rescued by the S2′ site at the early infection stage. This may be attributed to a trade-off between cell-cell fusion and production of cell-free virions. Enhanced S2′-mediated membrane fusion promotes direct viral spread between adjacent cells, which can efficiently propagate infection without requiring the release of free progeny virions. While this strategy facilitates rapid local dissemination and exacerbates cytopathology, it may also accelerate host cell death and reduce the overall yield of infectious particles released into the supernatant. This phenomenon has been described for other fusogenic viruses such as respiratory syncytial virus, where enhanced fusion activity correlates with increased pathogenesis but not necessarily with higher cell-free virus titers (36).

Our *in vivo* experiments in 1-day-old SPF chickens confirmed the significant attenuation of S1/S2 cleavage site-deficient IBV strains. This was evidenced by milder clinical symptoms, lower mortality rates, and alleviated tissue damage (Fig. 3 to 5). qPCR and immunohistochemistry consistently demonstrated that deletion of the S1/S2 site significantly reduced viral RNA loads and antigen in primary target organs (trachea and kidney) (Fig. 6 and 7). These findings underscore the critical role of the S1/S2 cleavage site in IBV pathogenicity and highlight its potential as a target for rational attenuation in live attenuated vaccine development. This approach is supported by studies on SARS-CoV-2, where deletion of the S1/S2 MBCS (ΔPRRA) reduced viral replication in Calu-3 cells and significantly alleviated weight loss in the hamster challenge models, while still permitting efficient replication for immune response elicitation (31). Meanwhile, a striking observation from our *in vivo* studies was the different pathogenicity of rYN and rM41 MBCS mutants. rM41 and its mutants primarily caused respiratory lesions with low mortality, whereas rYN and its derivatives, particularly rYN-S2′, induced more severe renal and neurological damage, culminating in significantly higher mortality (Fig. 3 and 4). This disparity aligns with known pathological profiles of respiratory (Mass-like) and nephropathogenic (QX-like) IBV strains (37) and suggests fundamental differences in their tissue tropism and virulence. The Mass-like IBV strain was the first to be isolated in the 1930s, which is typically known for inducing respiratory symptoms (38). The mortality rate of M41-infected chickens was very low, and is mainly related to secondary infection with bacteria (39). While the QX-type virus has a relatively broad tissue tropism with a preference for urogenital organs as well as lymphoid organs in birds, the bursa of Fabricius (11). When IBV acquires a wider range of tissue tropism such as the genitourinary tract and neurotropism, its mortality rate increases significantly (17, 40).

The introduction of a furin cleavage motif at the S2′ position has been shown in other coronaviruses to broaden entry pathways. A study by Le Coupanec et al. demonstrated that cleavage of the HCoV-OC43 spike protein at both the S1/S2 and S2′ sites is essential for its neuroinvasiveness. Particularly, mutations at the S2′ site (KASSRS→KASSAS) significantly impaired the virus’s ability to enter and spread in neuronal cells (41). The study further demonstrated that mutations at different spike protein cleavage sites alter the virus’s dependence on host proteases (such as cathepsin B and TMPRSS5), thereby influencing its entry pathways into neural cells. This suggests that viruses can adapt to diverse cellular environments by modifying their cleavage strategies. For SARS-CoV, an engineered S2′ cleavage site enabled utilization of a cell surface pathway in addition to the endosomal route (42). Furthermore, the S2′ cleavage site in SARS-CoV-2 influences spike protein processing and confers an adaptive advantage in certain cell types (35). In alignment with these observations, our study confirms that the presence of an S2′ cleavage site enhances neurological organ-specific tropism in both the rYN and rM41 genetic backgrounds. Both rYN-S2′ and rM41-S2′ infected chickens exhibited markedly increased viral loads in neural tissues (cerebellum, cerebrum, and sciatic nerves) compared to their parental strains and S1/S2-deficient mutants (Fig. 6). IHC confirmed viral antigen in the cerebrum (Fig. 7). Notably, the presence of the native S1/S2 cleavage site significantly amplified the neurodissemination and virulence mediated by the S2′ site (compare rYN/rM41-S2′ with rYN/rM41-ΔS1/S2-S2′), suggesting a synergistic interaction between the two cleavage sites.

Coronaviruses have evolved multiple strategies to exploit host proteases for spike protein cleavage activation, and the repertoire of proteases available in target cells critically influences viral tropism and pathogenesis (43). In coronaviruses possessing the S1/S2 MBCS like SARS-CoV-2, the S protein is efficiently cleaved by furin, and this cleavage is essential for S-protein-mediated cell-cell fusion and entry (5, 6). However, several other proteases, such as TMPRSS2, MMP2/9, cathepsins, and neutrophil elastase, have also been suggested to cleave the SARS-CoV-2 S1/S2 site (44), which is supported by the observation that furin knockout does not fully prevent cleavage of the S1/S2 site (45). For coronaviruses lacking a canonical MBCS at S1/S2, such as SARS-CoV, MERS-CoV, and HCoV-229E, spike protein activation is achieved through cleavage by alternative host proteases including trypsin, cathepsins, or TMPRSS family proteases during entry (46). Bioinformatic analysis of the S protein sequences from prevalent IBV strains revealed a conserved motif—YVSYG(K/R) (F/Y) C↓I(K/E/Q) PDG—located approximately 10 amino acids downstream of the S1/S2 site, which is predicted to be a CTSL cleavage motif. This predicted motif may explain why, even after mutating the native S1/S2 MBCS (RRFRR) to a non-cleavable sequence (AAFAA), residual cleaved S1/S2 products remained detectable by western blot (Fig. 1D). At the same time, this also explains why the virus remains capable of entry and replication even after the S1/S2 MBCS has been mutated.

Notably, different SARS-CoV-2 variants exhibit distinct protease usage. The Delta variant predominantly employs metalloproteinases for cell entry, whereas the Omicron variant has shifted its dependency toward cathepsin B/L while becoming less efficient in utilizing TMPRSS2, a phenomenon linked to specific spike mutations (47–51). This variant-specific plasticity highlights the dynamic nature of protease-dependent entry among coronaviruses. To experimentally dissect protease dependence in different IBV strains, we used specific inhibitors targeting furin (MI-1851), TMPRSS2 (camostat), and CTSL (E64D). As shown in Fig. 8B and C, furin-mediated cleavage at S1/S2 was not essential for replication, as rM41, rYN, and their ΔS1/S2 mutants were not sensitive to the furin inhibitor MI-1851, indicating that the lack of furin can be compensated by other proteases such as TMPRSS2. In contrast, the S2′ cleavage site was heavily dependent on furin activity, as MI-1851 significantly suppressed the replication of mutants carrying the S2′ site, with the inhibitory effect primarily observed at 12 hpi. This indicates that the effect of the S2′ MBCS on viral replication is indeed mediated by furin protease. As shown in Fig. 9A and B, all IBV strains relied heavily on the TMPRSS2-mediated serine protease pathway, as camostat dramatically reduced viral replication, confirming that TMPRSS2 usage is a common requirement for efficient IBV entry into CEK cells, regardless of the specific MBCS composition. We also noticed the increased sensitivity of S1/S2-deleted IBV mutants to camostat, indicating that loss of the S1/S2 site shifts the dependency toward TMPRSS2-dependent entry. This is consistent with a report on SARS-CoV-2, where TMPRSS2 was found to directly cleave S1/S2-mutant peptides and, in the complete absence of S1/S2 priming, enhanc cell-to-cell fusion by promoting S2′ cleavage and releasing an S1L fragment (52). This suggests that when the native S1/S2 multibasic cleavage site is disrupted, the virus may become more reliant on TMPRSS2-mediated activation as an alternative entry pathway. However, the two strains diverged in their utilization of the CTSL-mediated endosomal pathway. rM41 showed minimal dependence on cathepsins, with E64D having no effect on its early replication at 12 hpi. In contrast, rYN could utilize the cathepsin-mediated pathway, as E64D treatment significantly reduced its viral loads at 12 hpi. Notably, the inhibitory effects of both camostat and E64D on viral replication gradually diminished at later time points (36 hpi), likely because these inhibitors primarily target the early steps of virus entry and replication, and their impact becomes masked by subsequent rounds of viral propagation in the highly permissive CEK cell system. Moreover, cleavage site modifications altered protease dependence: mutation of the S1/S2 site in rYN (rYN-ΔS1/S2) substantially reduced its reliance on CTSL, while the same mutation in rM41 (rM41-ΔS1/S2) unexpectedly increased its dependence on this pathway. The S protein cleavage patterns were also distinct between these two virus backbones (Fig. 9C). Although the inhibitor-associated changes in S cleavage patterns observed by Western blot were not sufficiently precise to define protease-specific cleavage events, they may still provide a preliminary indication that alterations in the MBCS can influence protease responsiveness of the S protein. Previous studies have demonstrated that precleaved MERS viruses use receptor-proximal, cell-surface proteases (such as TMPRSS2) to effect the second fusion-activating cleavages during cell entry, whereas the more rigid uncleaved MERS viruses are routed past these cell-surface proteases and into endosomes, where they depend on cathepsins for activation (53). This established model provides a plausible explanation for the increased cathepsin reliance observed in rM41-ΔS1/S2 and rM41-ΔS1/S2-S2′. But for the rYN cleavage site mutants, the situation seems much more complicated. One possibility is that compensatory cleavage by other host proteases may differ between the two genetic backgrounds. It has been shown that the late lineages of SARS-CoV-2 evolved to more efficient use of the TMPRSS2-mediated entry pathway and gradually lost the ability to employ the cathepsins/endosome-mediated entry (54). Additional experiments, such as cathepsin-specific knockout or overexpression studies, will be required to fully elucidate the mechanistic basis of the differential cathepsin dependence between rYN and rM41.

Based on these findings, we propose a proteolytic coordination model for IBV S protein activation: the S1/S2 cleavage site primarily confers efficient replication competence, priming the S protein for subsequent activation steps and enabling effective receptor binding and structural rearrangements. Within this permissive context established by S1/S2 cleavage, the S2′ site then acts as an amplifier of organ-specific pathogenicity, particularly enhancing neurotropism. This dual cleavage mechanism would promote more efficient viral entry and dissemination, accounting for the severe neuropathology observed in the rYN/rM41-S2′ strain. Consequently, the presence of a MBCS at S2′ not only alters cell entry pathway preferences but, when coupled with a functional S1/S2 site, can dramatically reshape the disease outcome by directing the virus to new tissue niches. The insertion of multibasic cleavage sites, as exemplified by the S2′ site in Beaudette and our engineered strains, represents a significant evolutionary mechanism for coronaviruses to alter tropism and enhance virulence. Our findings highlight the importance of monitoring the emergence of such insertions in circulating IBV strains and other coronaviruses, as they may signal increased zoonotic potential or heightened pathogenicity. Future work should focus on elucidating the precise host proteases that cleave the predicted alternative CTSL site in IBV S protein and investigating the detailed structural changes in the S protein induced by sequential cleavage at the S1/S2 and S2′ sites.

## MATERIALS AND METHODS

### Animals

All specific-pathogen-free (SPF) embryonated chicken eggs (ECEs) and SPF chickens used in this study were procured from Beijing Boehringer Ingelheim Vital Biotechnology Co., Ltd. (Beijing, China).

### Cell culture and virus strains

Primary chicken embryo kidney cells (CEK) were derived from 18-day-old SPF ECEs. Baby hamster kidney cells (BHK-21) were cultured in Dulbecco’s modified Eagle’s medium (DMEM, Thermo Fisher Scientific, Waltham, MA, USA), supplemented with 10% fetal bovine serum, 100 units/mL penicillin, and 100 μg/mL streptomycin (Gibco, USA). All cell cultures were maintained at 37°C in a 5% CO_2_ atmosphere. The Beaudette (GenBank: MZ368698.1), M41 (GenBank: DQ834384.1), and YN (GenBank: JF893452.2) strains were preserved in our lab and propagated in 10-day-old SPF embryos, serving as the parental virus in all experiments.

### Generation of mutant viruses

Virus rescue experiments were conducted as previously described (19). In brief, confluent monolayers of BHK-21 cells (10^6^ cells per well in six-well plates) were transfected with 2.5 μg per well of the IBV YAC-BAC shuttle plasmid using Lipofectamine 2000 (Thermo Fisher Scientific). Following a transfection period of 6 h, the transfection medium was replaced with post-infection medium (DMEM supplemented with 2% FBS and 1% PSG). After 48 h post-transfection, the cells were cryopreserved at −80°C and designated as passage 0 (P0). Cells underwent three freeze/thaw cycles before the lysates were inoculated into the allantoic cavities of 9- to 11-day-old SPF chicken embryos. Allantoic fluid was harvested 48 h post-inoculation for further passages.

### Virus growth kinetics

Virus growth kinetics were analyzed in both CEK cells and ECEs. The CEK cells in 24-well plates were infected with 10^3^ TCID_50_ of IBV. After 1 h of adsorption at 37°C, surface virions were removed by washing the cells three times with PBS. To evaluate replication curves in ECEs, 10-day-old embryos were inoculated with 10^3^ TCID_50_ of IBV in 0.1 mL via the allantoic cavity. Cell supernatants or allantoic fluids were collected at 6, 12, 24, 36, 48, and 60 hpi. Viral titers were determined on CEK cells via the Reed-Muench method based on cytopathic effects observed 5 days post-infection with 10-fold serial dilutions.

### Western blot

CEK cells were infected with 10^7^ copies of recombinant virus. After a 1-h incubation at 37 °C, the inoculum was removed, and the cells were washed with PBS. DMEM with 2% FBS was then added. At 36 hpi, the supernatant was discarded, and the cells were washed again. Total cellular protein was extracted using radioimmunoprecipitation assay (RIPA) buffer, mixed with loading buffer, and boiled for 10 min. Proteins were separated by SDS-PAGE and transferred onto a PVDF membrane. The membrane was blocked with 5% non-fat milk for 1 h at room temperature and washed three times with PBST (PBS containing 0.1% Tween-20). The primary antibodies employed include a mouse monoclonal antibody against the N protein (diluted 1:1,000 in PBS; HyTest, Turku, Finland), a mouse monoclonal antibody against the S2 protein (prepared in-house, diluted 1:5,000 in PBS), and an anti-GAPDH antibody as an internal control (mouse monoclonal, CST #97166). After overnight incubation at 4°C, the membrane was washed three times with PBST and incubated with a HRP-conjugated goat anti-mouse secondary antibody (diluted 1:3,000 in PBS; CST, USA) for 1 h at room temperature. Finally, ECL substrate was applied, and the signals were visualized using a multifunctional imaging system (ChemiDoc MP, Bio-Rad).

### Indirect immunofluorescence (IF) assay

CEK cells were cultured in 24-well plates and subsequently infected with recombinant IBV. The cells were washed with PBS and fixed with 4% paraformaldehyde for 20 min at room temperature. After fixation, the cells were permeabilized with 0.2% Triton X-100 in PBS for 10 min and then blocked with 5% bovine serum albumin (BSA) for 1 h at room temperature. The fixed cells were incubated with a mouse monoclonal antibody against the IBV N protein (diluted 1:1,000 in PBS; HyTest, Turku, Finland) overnight at 4°C. After three washes with PBS, the cells were incubated with Alexa Fluor 555-conjugated goat anti-mouse IgG secondary antibody (CST, #8953) for 1 h at room temperature in the dark. Nuclei were counterstained with 4′,6-diamidino-2-phenylindole (DAPI; CST, #4083) for 5 min. Following the three final PBS washes, the cells were imaged using a fluorescence microscope.

### Animal experiment design

A total of 180 one-day-old specific-pathogen-free (SPF) chickens were randomly divided into nine groups (*n* = 20 per group). Each group was inoculated with 100 μL of rYN, rYN-ΔS1/S2, rYN-S2′, rYN-ΔS1/S2-S2′, rM41, rM41-ΔS1/S2, rM41-S2′, and rM41-ΔS1/S2-S2′ (each at 10^5^ EID_50_) via the ocular route. The control group received 100 μL of sterile PBS as a negative control. All chickens were provided with *ad libitum* access to food and water throughout the 14-day observation period. Clinical signs were monitored daily. On days 3, 5, 7, and 14 post-infection, three chickens from each group were randomly selected for necropsy and pathological examination.

#### Clinical observation

Throughout the 14-day observation period, chickens were monitored daily for clinical signs and scored according to the following criteria: 0 = no clinical signs; 1 = mild depression, rales, watery droppings, slight head shaking, hunched posture, or lacrimation; 2 = sneezing, coughing, marked depression, watery droppings, or nasal discharge; 3 = open-mouth breathing, neurological symptoms, or severe watery droppings; and 4 = moribund state or death.

#### Ciliary activity scoring of the trachea

The trachea of each chicken was excised and divided into upper, middle, and lower sections. From each section, three to four complete tracheal rings approximately 1 mm in width were collected, totaling 10 rings per bird. The tracheal rings were immediately placed into a 96-well culture plate containing 100 μL of DMEM per well. Ciliary movement was examined under a light microscope, and the extent of ciliary activity was scored based on the following criteria: 0 = 100% ciliary activity; 1 = 75–100% ciliary activity; 2 = 50–75% ciliary activity; 3 = 25–50% ciliary activity; and 4 = 0–25% ciliary activity.

#### Histopathology

Tissue samples were processed following standard paraffin embedding procedures, including dehydration, clearing, paraffin infiltration, and embedding, sectioning, water bath spreading, and slide drying. The paraffin sections were then stained with hematoxylin and eosin (H&E) and mounted. Histopathological evaluation was performed under a light microscope.

#### Immunohistochemistry

Fixed samples were processed, embedded in paraffin wax, and cut into 5-μm sections for immunohistochemistry (IHC). To block nonspecific binding, tissue sections were first incubated with goat serum diluent. A mouse monoclonal antibody against the IBV N protein (diluted 1:1000 in PBS; HyTest, Turku, Finland) was then applied, followed by the addition of 50 μL of signal amplification reagent. Goat anti-mouse IgG was used as the secondary antibody.

### Cell viability assay

The effects of E64D, camostat, and MI-1851 on cell viability were measured by the CCK-8 assay. CEK cells were seeded into a 96-well plate and allowed to adhere until the cells were about 70% confluent, followed by treatment with different concentrations (0.1, 0.1, 0.5, 1, 5, and 10 μM) of drugs or the equivalent amount of DMSO for 24 h. The cells without any treatments were used as the blank control. After treatments, CCK-8 was added to each well, and the cells were cultured for an additional 1 h. A microplate reader (BD AriaIII) was used to determine the absorption at the wavelength of 450 nm, and the inhibition was calculated as: Inhibition rate% = [(control group blank) − (Drug treatment group blank)]/(control group blank) × 100%. The average absorbance reflected cell viability with the data normalized to the blank control group. Experiments were done in quintuplicates and repeated at least three times.

### Protease inhibitor treatment assay

The cathepsin inhibitor, Aloxistatin (E64D) (HY-100229), the serine protease inhibitor, camostat (HY-13512), and the furin protease inhibitor, MI-1851 (HY-150737) were purchased from MedChemExpress (Monmouth Junction, NJ, USA). CEK cells were treated with DMSO, E64D, camostat, or MI-1851 at concentrations of 10 µM for 2 h before infection with either rYN-based MBCS mutant IBVs and their parental strain rYN, or rM41-based MBCS mutant IBVs and their parental strain rM41. After 12 h, a subset of the cells was washed, fixed with methanol, and processed for IFA as described above. At 12, 24, and 36 hpi, the remaining cell lysates and supernatants were collected for qRT-PCR quantification of virus replication. For proteinase cleavage assays, 293T cells were treated with E64D or camostat for 2 h before being transiently transfected with plasmids (pcDNA3.1-rM41-S and pCAGGS-rYN-S) expressing S proteins of rM41 and rYN. At 24 h post-transfection, the 293T cells were harvested to analyze S protein cleavage patterns, with the S2/S0 ratio quantified using ImageJ software.

### Quantitative reverse transcription-PCR (RT-qPCR)

Viral RNA was extracted using the HiPure RNA minikit (Magen, Beijing, China), reverse-transcribed with the StarScript III All-in-one RT Mix with gDNA Remover kit (Genstar, Beijing, China), and quantitative real-time PCR was conducted using the Light Cycler 96 with 2× M5 Hiper SYBR Premix EsTag (Mei5bio, Beijing, China). The primers were designed targeting the conserved 5′UTR of IBV (qpF: 5′-GTTGGGCTACGTTCTCGC-3′, qpR: 5′-AAGCCATGTTGTCACTGTCTAT-3′). Copy numbers (*x*) were calculated from Ct values based on a standard curve (Ct = −3.41 × log10*x* + 39.1).

### Statistical analysis

Statistical analysis was performed using GraphPad Prism 9.0. Two-way analysis of variance (two-way ANOVA) and the Student’s *t*-test were performed to conduct statistical analysis. Data are expressed as the mean ± standard deviation (SD). *P* values < 0.05 were considered statistically significant and denoted by *P < 0.05, **P < 0.01, ***P < 0.001, and ****P < 0.0001. All experiments in which statistical tests were used were repeated independently at least three times.

## Data Availability

The main data supporting the findings of this study are available from the corresponding author upon reasonable request.
